# Inter-individual variations of human mercury exposure biomarkers: a cross-sectional assessment

**DOI:** 10.1186/1476-069X-4-20

**Published:** 2005-10-03

**Authors:** Marika Berglund, Birger Lind, Karolin Ask Björnberg, Brita Palm, Östen Einarsson, Marie Vahter

**Affiliations:** 1 Department of Metals and Health, Institute of Environmental Medicine, Karolinska Institutet, Box 210, SE-171 77 Stockholm, Sweden; 2 Analys Modul Sweden AB, Tingsvägen 19, SE-191 61 Sollentuna, Sweden

## Abstract

**Background:**

Biomarkers for mercury (Hg) exposure have frequently been used to assess exposure and risk in various groups of the general population. We have evaluated the most frequently used biomarkers and the physiology on which they are based, to explore the inter-individual variations and their suitability for exposure assessment.

**Methods:**

Concentrations of total Hg (THg), inorganic Hg (IHg) and organic Hg (OHg, assumed to be methylmercury; MeHg) were determined in whole blood, red blood cells, plasma, hair and urine from Swedish men and women. An automated multiple injection cold vapour atomic fluorescence spectrophotometry analytical system for Hg analysis was developed, which provided high sensitivity, accuracy, and precision. The distribution of the various mercury forms in the different biological media was explored.

**Results:**

About 90% of the mercury found in the red blood cells was in the form of MeHg with small inter-individual variations, and part of the IHg found in the red blood cells could be attributed to demethylated MeHg. THg in plasma was associated with both IHg and MeHg, with large inter-individual variations in the distribution between red blood cells and plasma. THg in hair reflects MeHg exposure at all exposure levels, and not IHg exposure. The small fraction of IHg in hair is most probably emanating from demethylated MeHg. The inter-individual variation in the blood to hair ratio was very large. The variability seemed to decrease with increasing OHg in blood, most probably due to more frequent fish consumption and thereby blood concentrations approaching steady state. THg in urine reflected IHg exposure, also at very low IHg exposure levels.

**Conclusion:**

The use of THg concentration in whole blood as a proxy for MeHg exposure will give rise to an overestimation of the MeHg exposure depending on the degree of IHg exposure, why speciation of mercury forms is needed. THg in RBC and hair are suitable proxies for MeHg exposure. Using THg concentration in plasma as a measure of IHg exposure can lead to significant exposure misclassification. THg in urine is a suitable proxy for IHg exposure.

## Background

People are exposed to different forms of mercury (Hg), which differ with respect to toxicology. The target organ for methylmercury (MeHg) toxicity is the brain, which is especially susceptible during development [1]. Target organs for elemental mercury vapour (Hg^0^) are the brain and kidney and the target organ for inorganic Hg compounds (IHg, Hg^2+^) is the kidney [1]. Both MeHg and Hg^0^, but not IHg, readily passes the blood-brain and placental barriers [1]. Exposure to MeHg occurs almost exclusively via consumption of seafood, especially predatory fish and large marine mammals, while food in general contains low concentrations of both MeHg and IHg [2-4]. Dental amalgam fillings, releasing Hg^0^, are the major source of Hg^0 ^exposure in the general population [5].

In the gastrointestinal tract MeHg is absorbed to approximately 95% [6,7], Hg^2+ ^to approximately 7% [8] and elemental Hg to less than 0.01% [9,10]. The absorption of Hg^0 ^in the lung is about 80% [11]. Within tissues, MeHg is slowly demethylated to Hg^2+ ^[12,13]. In the blood, Hg^0 ^is readily oxidized to Hg^2+ ^by catalase [14].

The total mercury concentration (THg) in blood is often used as a proxy measure of MeHg exposure in individuals eating fish with the assumption that the IHg exposure, and thereby the IHg concentration in blood, is much lower [15-18]. In the blood, more than 90% of MeHg is bound to haemoglobin in the red blood cells (RBC), while IHg is more evenly distributed between RBC and plasma [7,19]. Therefore, total Hg in RBC is also sometimes used as a proxy measure of MeHg exposure [20-23] and total Hg in plasma is used as a proxy measure of IHg exposure (Hg^2+ ^and Hg^0^; [3,22,24-26]).

The concentration of total Hg in hair (H-THg) is often used as a measure of MeHg exposure, assuming that > 80% of Hg in hair is in the form of MeHg [27]. Mercury is incorporated in hair during formation in the hair follicle, and mercury in hair is associated with the concentration of MeHg in blood [19]. It has been proposed that H-THg reflects inorganic mercury exposure at low MeHg exposure in populations with no or low fish consumption [1].

The total Hg concentration in urine is used as a measure of IHg exposure as MeHg is excreted primarily via the bile (as glutathione complex) and faeces (about 90%; as IHg) and only to a limited extent (about 10%) in urine (as IHg; [1,3,28]).

The mercury biomarkers are frequently used for estimation of exposure and risks of health effects, but the inter-individual variations are not well known. The available information on Hg kinetics is based on 25–35 years old experimental studies, sometimes with high exposure levels, involving a limited number of volunteers. The aim of the present study was to investigate the robustness of some of the statements forming the basis for the biomarkers frequently used, and to explore the inter-individual variations. In order to do so, we have improved the traditional Magos' mercury speciation method [29,30] and developed an automated analytical system for speciation of mercury in whole blood, RBC, plasma, hair and urine.

## Methods

### Sampling

In 2003 we recruited 28 volunteers, 23 women and 5 men, 28–60 years of age (mean 48 years) for measurement of Hg biomarkers. Sampling comprised venous blood from the cubital vein (5 mL, Venoject II, EDTA(K2), VP-050SDK), red blood cells (RBC) and plasma (5 mL, Venoject II, EDTA(K2), VP-050SDK; Terumo Corp., Leuven; Belgium), hair (a hair sample was tied with a cotton thread, cut close to the scalp from the back of the head and put into a plastic bag), and urine (a spot sample collected in acid washed plastic containers). Information regarding fish intake (usual number of meals/month) and number of dental amalgam fillings was collected via self-reported questionnaires. A usual number of 0–22 fish meals/months and a total number of dental amalgam fillings between 0–15 were reported. For evaluation of Hg distribution in hair, and blood to hair ratio, we also used data previously collected in a study of women with a high fish intake (N = 145, 20–50 years of age; [31]). The study was approved by the Ethics Committee of the Karolinska Institutet, Stockholm.

### Sample treatment

Whole blood, RBC, plasma and urine samples (1.0 mL) were treated with 1.0 mL L-cysteine (0.012 M), 1.5 mL NaOH (11 M) and 0.5 mL deionised water, and stored in the dark over night at room temperature to complete the solubilisation. Hair samples (3 cm from the scalp end; approximately 20 mg) were treated with 2.0 mL L-cysteine (0.083 M), 4.0 mL NaOH (11 M) and 14 mL NaCl (0.17 M). The mixture was heated to 90–95°C for 20 minutes to complete the solubilisation.

### Analyses

**Figure 1:**
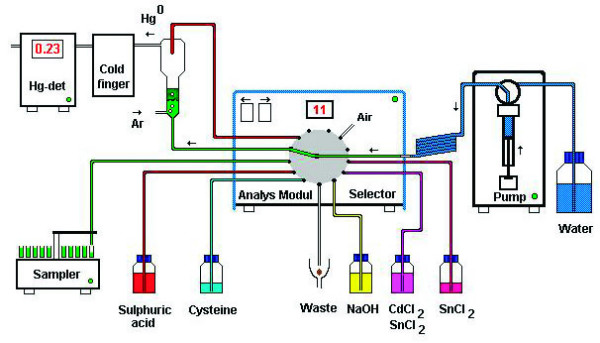
**The automatic mercury analysis system**. The automatic multiple-injection analysis (MIA) system, with a Tefzel 13-channel selector valve (Analys Modul Sweden AB) for mercury analysis.

Total mercury (THg) and inorganic Hg (IHg) were analyzed in whole blood, RBC, plasma, hair, and urine using cold vapour atomic fluorescence spectrophotometry (CVAFS, Merlin, PSA 10.023; P.S. Analytical Ltd., Orpington, Kent, UK), following reduction to Hg^0 ^in a reaction tower, using an automatic multiple-injection analysis (MIA) system, with a Tefzel^® ^13-channel selector valve (Analys Modul Sweden AB; Figure 1). In this system, a motor-driven pump (Microlab 900, Hamilton Bonaduz AG, Switzerland) is connected to the central port of the selector valve, which opens to one of 13 peripheral ports at a time. The pump dispenses volumes with low variation which enables low volumes of chemicals to be used. Deionised water is used as an extended syringe piston and washing medium. No chemicals or samples reach the syringe at the base of the tube loop (Figure 1). The sampler, the reaction tower and the reagent bottles are connected to the peripheral ports by Tefzel^® ^tubes. The sampler and the selector, with an AD converter, are controlled by a PC using a control program (EASYLAB, Analys Modul Sweden AB). The program executes the commands from a command list for THg or IHg in a sequential or logical order. In order to reduce blank values, all reagents are initially mixed in the reaction tower to eliminate any Hg impurities. Any Hg^0 ^formed in this initial cleaning step is transported with argon (Ar) gas through the detector.

For determination of IHg, 800 μl L-cysteine solution (0.1% w/v L-cysteine in 1.5% w/v NaCl), 200 μl 8 M H_2_SO_4 _(p.a.) with 0.4% antifoaming agent (Antifoam 204; Sigma Chemical Co., S:t Louis, MO, USA; soluble in acid but not in alkaline solution), 2000 μl 11.25 M NaOH, 100 μl deionised water, and 100 μl 10% w/v SnCl_2 _in 2.4 M H_2_SO_4 _were delivered to the reaction tower for elimination of Hg impurities, after which 500 μl sample solution and 200 μl cysteine solution, followed by 1200 μl of deionised water (for rinsing) were added. For determination of THg, 800 μg L-cysteine solution, 600 μl 8 M H_2_SO_4 _with 0.4% antifoaming agent, 2000 μl 11.25 M NaOH, 200 μl cysteine solution, 1000 μl deionised water, and 100 μl of a mixture of 10% CdCl_2 _and 50% SnCl_2 _in 8 M H_2_SO_4 _were delivered to the reaction tower for elimination of Hg impurities. Then 500 μl 8 M H_2_SO_4 _(supra pure) was added (in order to increase the temperature), followed by 500 μl sample solution and 500 μl deionised water.

The Hg^0 ^released from the sample was transported by Ar gas (0.087 L/min) through a moisture trap, chilled with ice, followed by a tubular permeable membrane (Perma Pure mini-dryers, model MD-125-12S, Perma Pure Products, Inc, Farmingdale, USA) before reaching the AFS detector. The signal was stored by the PC and also recorded on paper for process control (Perkin Elmer Model 56 recorder). The area under the curve was integrated by the computer and used for evaluation of the amount (ng) Hg in the sample. The shield gas flow (Ar) for the detector was 0.099 L Ar/min. The standard solutions (0.1–2.0 ng Hg/mL) were made in 0.1% L-cysteine. Furthermore, a MeHg standard of 0.4 ng/mL was included in the standard curve in order to control the degree of demethylation in the IHg analysis and for recovery in the THg analysis. The time interval between reactions was 15 minutes. All samples were analyzed in duplicates.

The concentrations of the organic mercury fraction (OHg) were calculated by the subtraction of the IHg concentrations from that of the THg concentrations. OHg is assumed to be mainly MeHg as the only other known exposure sources of organic mercury compounds in Sweden are a few vaccines containing thiomersal (ethylmercurithiosalicylate), a seldom-used preservative containing ethylmercury. Hg concentrations in urine were adjusted to specific gravity (1.019 μg/mL; urine specific gravity refractometer, Uricon-Ne, Atago Co., Ltd., Tokyo, Japan) and creatinine (analyzed at the Department of Clinical chemistry, Karolinska University Hospital, Stockholm). Haematocrit and haemoglobin (Hb) concentrations were measured (Department of Clinical chemistry, Karolinska University Hospital, Stockholm).

### Evaluation of MeHg demethylation during sample treatment and analysis

For evaluation of potential demethylation during solubilisation, a purified (> 99%) radiolabeled methylmercury-chloride (^203^Hg, Amersham Laboratories, Amersham, UK) solution was solubilised according to the method described above, at room temperature and at 88°C for one hour. The total Hg concentration was measured by gamma-counting (Searle 1195, Searle Analytical Inc.). The loss of total Hg during solubilisation was < 1%, i.e. not detectable. An aliquot (10 g) of each of the solubilised solutions was acidified by 3 mL 6 M HCl, stored over night at +4°C, extracted with chloroform three times (30+20+10 mL) to separate MeHg and IHg [32]. The water phase, containing the IHg from MeHg demethylation, was then measured by gamma-counting to calculate the degree of demethylation during solubilisation.

In order to quantify the degree of demethylation in the reaction tower during the analytical step, samples with and without addition of IHg and MeHg were solubilised and then acidified and extracted with chloroform, following the procedure described above. THg and IHg were then measured by the analytical method described above, and IHg from demethylation of MeHg was calculated as the percentage of the initial amounts.

### Analytical quality control

The blood sampling material was tested for Hg contamination. Simulated blood sampling using a weak acid (0.03 M HNO_3_) was performed. The acid solutions were analyzed for Hg content by the method described above. The material was found to be essentially free from Hg contamination (all acid solutions were below the limit of detection, LOD, i.e. 3 standard deviations of the mean of the chemical blanks). All other materials used for analysis were acid washed. Appropriate reference materials for Hg in blood, serum, urine and hair were analyzed in each analytical run, respectively (see Results and Table 1).

**Table 1:** Results of analytical quality control. Results (Mean; standard deviation, SD; coefficient of variation, CV%; and number, n) of repeated analyses of total mercury (THg) and inorganic mercury (IHg) in reference materials analyzed together with collected samples of whole blood, red blood cells (RBC), plasma, urine (μg/L) and hair (mg/kg).

**Media**	**Reference material**	**THg**	**IHg**
		Mean (SD)	Mean (SD)
		CV% (n)	CV% (n)
**Blood/RBC**	**Seronorm 404107X**		
	Rec. value THg: 3 μg/L	2.3 (0.18)	0.54 (0.051)
	Range: 2.2–3.3 μg/L	8.1 (7)	9.4 (7)
	**Seronorm 404108**		
	Rec. value THg: 8 μg/L	8.1 (0.52)	6.4 (0.32)
	Range: 6.7–8.4 μg/L	6.3 (7)	5.0 (7)
**Plasma**	**Seronorm 201405**		
	Rec. value THg: 0.96 μg/L	1.0 (0.081)	0.70 (0.017)
	Range: 0.87–1.06 μg/L	8.0 (5)	2.4 (5)
**Urine**	**Seronorm 2524**		
	Rec. value THg: 0.21 μg/L	0.20 (0.0068)	0.11 (0.011)
	95% CI: 0.17–0.25 μg/L	3.4 (4)	10 (4)
	**Seronorm 2525**		
	Rec. value THg: 40.3 μg/L	38 (1.9)	38 (0.85)
	95% CI: 37.7–42.9 μg/L	5.0 (6)	2.2 (6)
**Hair**	**IAEA086**		
	Rec. value THg: 0.573 mg/kg	0.58 (0.028)	0.27 (0.022)
	95% CI: 0.534–0.612	4.8 (5)	8.1 (5)
	**IMM-hair^1^**		
	Rec. value THg: 4.8 mg/kg	4.7 (0.14)	0.50 (0.026)
	SD: 0.3 mg/kg	3.0 (5)	5.1 (5)

^1 ^Björnberg et al. 2003

### Statistics

We used spearman correlation (r_s_) test to test for associations between parameters, and linear regression analysis for evaluation of association between parameters when the requirements for normally distributed residuals were met. Statistical analyses were conducted using SigmaStat^® ^(Version 2.03 for Windows (Systat Software GmbH, Erkrath, Germany). Statistical significance was set to p < 0.05.

## Results

### Analytical method

The accuracy of the Hg speciation method, as evaluated by repeated analyses of reference materials, was satisfactory (Table 1). There are no commercially available reference materials for IHg. However the obtained values for IHg in blood were well in agreement with our results from previous analytical runs of the same Seronorm sample [31,33]. The analytical variability, as calculated by coefficients of variation (CV%) of duplicate analysis of collected samples and reference materials, was low (Table 2). The detection limits (LOD) were lowered significantly by the introduction of the cleaning step of the reagent chemicals in the reaction tower prior to the sample addition (Table 3). As a result, very few samples of whole blood (n = 3), plasma (n = 2), and RBC (n = 3) had IHg concentrations below LOD.

**Table 2:** Precision of duplicate analyses. Precision (Coefficient of variation, CV%) of duplicate analyses of total mercury (THg) and inorganic mercury (IHg) in blood, red blood cells (RBC), plasma, urine (μg/L) and hair (mg/kg), in collected samples and reference materials.

	**THg**			**IHg**		
	CV (%)	No. of duplicate analyses	Concentration range	CV (%)	No. of duplicate analyses	Concentration range

**Blood**	4.3	32	0.34–8.5	6.4	30	0.079–6.8
**RBC**	5.9	34	0.38–14	12	24	0.061–6.7
**Plasma**	5.3	29	0.048–1.3	7.3	26	0.060–1.1
**Hair**	2.6	32	0.081–4.9	9.0	31	0.01–0.52
**Urine**	3.2	35	0.19–6.2	2.5	34	0.094–6.3

**Table 3:** Concentrations of total, inorganic and organic mercury in various biological media. Concentrations of total mercury (THg), inorganic mercury (IHg) and organic mercury (OHg) in whole blood, plasma, red blood cells (RBC), urine and hair in 28 individuals, and limits of detection (LOD, i.e. 3 × standard deviation of mean of chemical blank/solubilisation solution; the number of samples was 5–10) of the two chemical runs.

		**THg**	**IHg**	**OHg**
**Whole blood (μg/L)**	Mean ± SD	2.2 ± 1.4	0.35 ± 0.23	1.8 ± 1.3
	Median	2.0	0.35	1.6
	Range	0.34–7.3	0–0.94	0.26–6.9
	LOD	0.05/0.09	0.03/0.06	
**Plasma (μg/L)**	Mean ± SD	0.65 ± 0.30	0.39 ± 0.26	0.26 ± 0.16
	Median	0.63	0.37	0.22
	Range	0.07–1.3	0–1.1	0.05–0.70
	LOD	0.04	0.02/0.05	
**RBC (μg/L)**	Mean ± SD	4.1 ± 2.6	0.29 ± 0.18	3.8 ± 2.5
	Median	4.0	0.26	3.6
	Range	0.40–14	0–0.70	0.25–13
	LOD	0.03/0.04	0.05/0.05	
**Urine (μg/L; adjusted to density 1.019)**	Mean ± SD	1.4 ± 1.2	1.4 ± 1.2	0.012 ± 0.073
	Median	1.0	1.0	0.015
	Range	0.27–6.1	0.18–6.3	0–0.11
	LOD	0.03/0.05	0.03/0.02	
**Urine (μg/g creatinine)**	Mean ± SD	1.9 ± 2.0	1.9 ± 2.1	0.013 ± 0.12
	Median	1.3	1.2	0.018
	Range	0.12–10	0.12–11	0–0.23
**Hair (mg/kg)**	Mean ± SD	0.76 ± 0.40	0.062 ± 0.030	0.69 ± 0.37
	Median	0.71	0.060	0.66
	Range	0.08–2.0	0.010–0.12	0.072–1.9
	LOD	0.01	0.01	

Demethylation of MeHg to IHg takes place during the solubilisation of samples, and in the reaction tower, during the analysis of the solubilised samples. After solubilisation at room temperature, acidification and extraction with chloroform, the percentage of excess IHg from demethylation of MeHg was 0.9 ± 0.1% (n = 4). The demethylation after solubilisation at 88°C was 2.6 ± 0.5% (n = 4). The acidification step, which is necessary in order to perform the extraction, probably also, increases to a small extent the degree of demethylation. In the reaction tower, the percentage of excess IHg (from further demethylation of MeHg) was calculated to 3.0 ± 0.3% (n = 4).

### Biomarker concentrations and correlations

A summary of the concentrations of Hg species in whole blood (B), red blood cells (RBC), plasma (P), urine (U) and hair (H) is given in Table 3. The correlations between the Hg species in the various media as well as the exposure variables fish consumption (number of meals per month; range 0–22 meals per month) and number of dental amalgam fillings (range 0–>15), are given in Table 4. Fish consumption was positively correlated with THg in blood (r_s _= 0.74, p < 0.001), RBC, and hair, and with OHg in blood, RBC, plasma and hair (Table 4). Fish consumption was also correlated with IHg in hair. Number of dental amalgam fillings was positively correlated with THg in plasma (r_s _= 0.46, p = 0.01) and urine (r_s _= 0.49, p = 0.009), and with IHg in blood, plasma and urine (Table 4). Mercury levels in blood (THg, IHg and OHg in whole blood, RBC or plasma) were not associated with haemoglobin and haematocrit.

**Table 4:** The Spearman correlation coefficients between mercury species in different media and exposure vavariables. Spearman correlation coefficients of inorganic mercury (IHg) and organic mercury (OHg) species in whole blood (B; μg/L), red blood cells (RBC; μg/L), plasma (P; μg/L) and urine (U: μg/L, adjusted to density 1.019) and IHg, OHg and total mercury (THg) in hair (H; mg/kg) and the exposure variables fish consumption (number of meals per month) and number of dental amalgam fillings. The number of samples are 25–28. The significance level is indicated as * p < 0.05; ** p < 0.01; *** p < 0.001.

	**B-OHg**	**RBC-IHg**	**RBC-OHg**	**P-IHg**	**P-OHg**	**H-THg**	**H-IHg**	**H-OHg**	**U-IHg**	**U-OHg**	**Fish**	**Amalgam**
**B-IHg**	0.19	0.83 ***	0.25	0.91 ***	0.05	0.28	0.29	0.27	0.81 ***	-0.11	0.07	0.48 *
**B-OHg**		0.38	0.96 ***	0.07	0.82 ***	0.87 ***	0.79 ***	0.87 ***	-0.07	0.27	0.82 ***	0.09
**RBC-IHg**			0.45 *	0.70 ***	0.34	0.46 *	0.42 *	0.43 *	0.73 ***	0.04	0.37	0.27
**RBC-OHg**				0.13	0.77 ***	0.82 ***	0.81 ***	0.81 ***	0.03	0.22	0.76 ***	0.14
**P-IHg**					-0.13	0.16	0.16	0.14	0.74 ***	-0.14	-0.04	0.49 *
**P-OHg**						0.77 ***	0.74 ***	0.75 ***	-0.003	0.29	0.82 ***	0.06
**H-THg**							0.86 ***	0.99 ***	0.18	0.32	0.75 ***	0.28
**H-IHg**								0.83 ***	0.28	0.28	0.63 ***	0.32
**H-OHg**									0.17	0.31	0.74 ***	0.30
**U-IHg**										0.05	-0.03	0.49 **
**U-OHg**											0.24	-0.007
**Fish**												0.13

### Mercury in blood

The distribution of OHg and IHg in whole blood between RBC and plasma was calculated as the percentage of total OHg (or IHg) in whole blood according to equation 1 and 2 (below), using individual haematocrit values (B-EVF, %). The range of B-EVF was 35–47% (mean 42%). Data below LOD were not included because of their uncertainty.

1) RBC-OHg * (B-EVF/B-OHg) * 100

2) P-OHg * ((1-B-EVF)/B-OHg) * 100

On average 87% of OHg in whole blood was localized in the RBC (95% CI of mean ± 3.7; range 76–104%; n = 20) and 9.6% in plasma (95% CI of mean ± 1.6; range 5.1–20%; n = 22). On average 34% of IHg in whole blood was localized in RBC (95% CI of mean ± 4.0; range 15–54%; n = 22) and 64% in plasma (mean; 95% CI of mean ± 5.4; range 30–81%; n = 22). The distribution of OHg or IHg between RBC and plasma did not change with increasing concentrations of the respective Hg form.

The concentration of IHg in RBC was positively correlated with both the concentration of OHg in RBC and the concentration of IHg in plasma (Table 4) indicating that IHg in RBC is a function of both IHg and OHg exposures. RBC-IHg was on average 6.8% of RBC-THg (median; range 3.3–24%), and increased with increasing concentrations of RBC-OHg (r_s _= 0.46; p = 0.03) and increasing consumption of fish (r_s _= 0.60; p = 0.003), but not with increasing number of dental amalgam fillings. In a person with no dental amalgam fillings RBC-IHg was 4.6% of RBC-THg.

**Figure 2:**
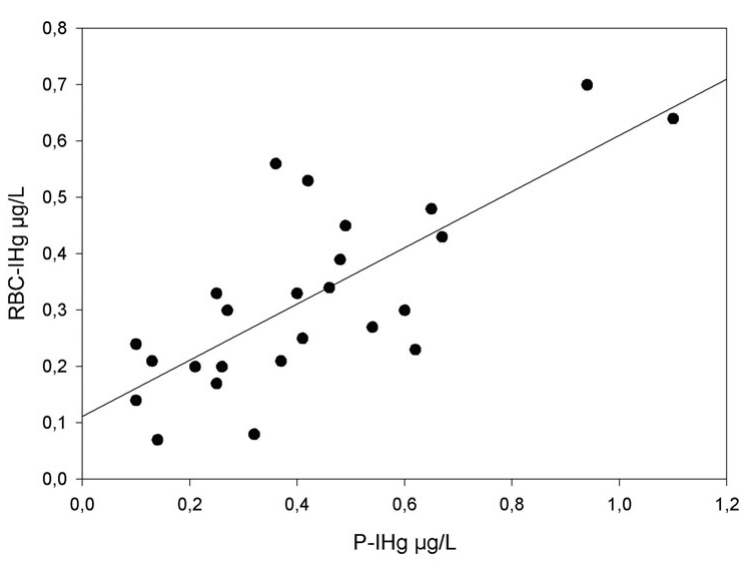
**The relationship between inorganic mercury in plasma and red blood cells**. The relationship between inorganic mercury in plasma (P-IHg) and red blood cells (RBC-IHg) evaluated by linear regression (RBC-IHg = 0.11+0.50 P-IHg; R^2 ^= 0.56).

The average RBC to plasma ratio of IHg concentrations was 0.90 (range 0.25–2.5), or as evaluated by linear regression, 0.50 (RBC-IHg = 0.11+0.50*P-IHg; R^2 ^= 0.56; Figure 2). The ratio increased with fish consumption (r_s _= 0.52; p = 0.008; n = 25), but not with the number of dental amalgam fillings (r_s _= -0.23).

The average RBC to plasma ratio of OHg concentrations was 14 (range 3.1–28). When evaluated by linear regression the ratio was also 14 (RBC-OHg = 0.20+14*P-OHg; R^2 ^= 0.72). The ratio did not increase with fish consumption (or number of dental amalgam fillings).

### Mercury in hair

The total mercury concentration in hair (H-THg) was positively correlated with B-OHg and P-OHg, as well as with fish consumption, but not with B-IHg or the number of dental amalgam fillings (Table 4). Speciation of Hg in hair showed that on average 91% of THg was OHg (CI of mean ± 1.2; range 79–95%; n = 28), and 8.9% was IHg (CI of mean ± 1.1; range 4.9–21%; n = 28). In our previous study of women with a high fish intake, the distribution was approximately the same, i.e. 91% of THg was OHg (CI of mean ± 1.2; range 82–97%) and 8.7% of THg was IHg (CI of mean ± 1.2; range 3.2–18%; n = 144). The percentage of IHg in hair was not associated with the number of dental amalgam fillings. The average percentage of IHg in hair was 8.3% (range 4.4–13%; n = 23) in individuals without dental amalgam fillings, and 8.8% (range 4.6–18%; n = 48) in individuals with 10 fillings or more (including data from our previous study). The difference was not statistically significant (Student's t-test, p = 0.4). The concentration of IHg in hair was highly correlated with OHg in hair, and with OHg in blood (B-OHg, RBC-OHg and P-OHg; Table 4), but not with IHg in blood (B-IHg, P-IHg or RBC-IHg; Table 4). The concentration of IHg in hair was also positively correlated with fish consumption, but not with number of dental amalgam fillings (Table 4).

**Figure 3:**
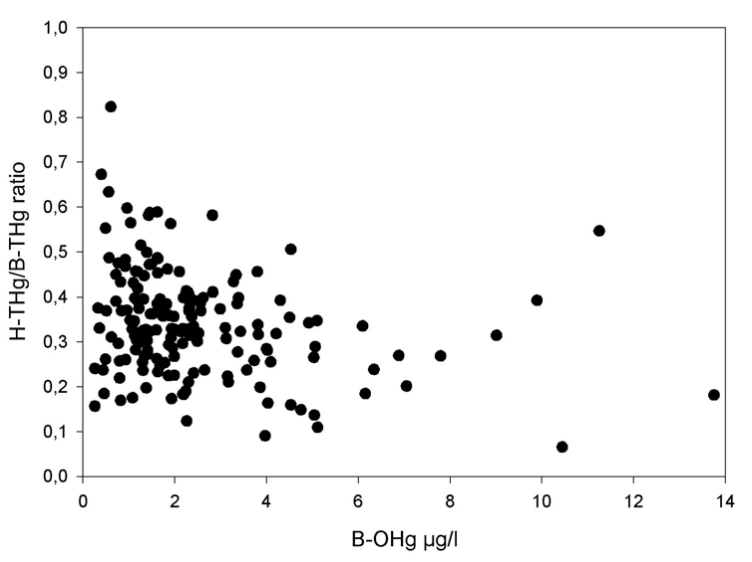
**The hair to blood ratio of total mercury versus organic mercury in blood**. The hair to blood ratio of total mercury (H-THg/B-THg) as a function of organic mercury in blood (B-OHg).

The average hair to blood ratio, (H-THg (mg/kg) divided by B-THg (μg/L)) was 0.366 (median 0.373; 95^th ^percentile 0.552; range 0.185 to 0.673). If H-THg was divided by B-OHg, the ratio was 0.465 (95^th ^percentile 0.670). If we included the hair and blood mercury data from our previous study of women with a high fish consumption the average hair to blood ratio was 0.341 (median 0.330; 5^th ^percentile 0.168; 95^th ^percentile 0.563; range 0.066–0.824; n = 173). The hair to blood ratio seemed to decrease with increasing B-OHg (Figure 3).

**Figure 4:**
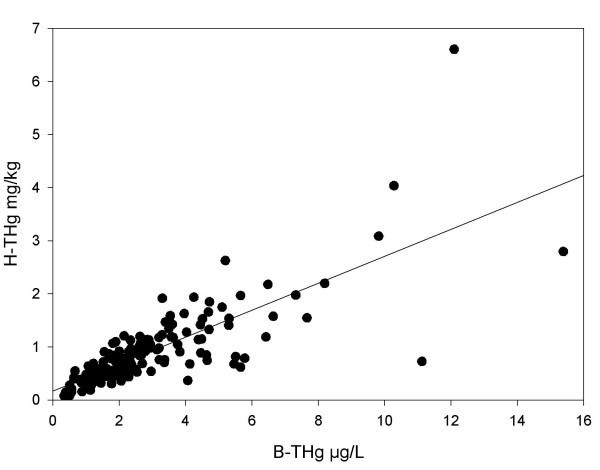
**The relationship between total mercury in hair and blood**. The relationship between total mercury in hair (H-THg) and blood (B-THg) evaluated by linear regression (H-THg = 0.169+0.254 B-THg; R^2 ^= 0.62).

The ratio as determined by linear regression of H-THg versus, B-THg was 0.264, i.e. H-THg = 0.179+0.264*B-THg (R^2 ^= 0.83; p < 0.001; n = 28). Replacing B-THg with B-OHg only resulted in an increased intercept, to 0.282 (R^2 ^= 0.80; n = 25). Inclusion of data from our previous study in the linear regression analysis resulted in H-THg = 0.169+0.254*B-THg (R^2 ^= 0.62; p < 0.001; n = 173; Figure 4).

### Mercury in urine

Essentially all Hg in urine (> 98%) was IHg. IHg in urine, adjusted to specific gravity (1.019 g/mL) or adjusted to creatinine (g creatinine/L urine) were highly correlated with IHg in blood, plasma and RBC, but not with OHg in the various media (Table 4). IHg in urine was moderately associated with the number of dental amalgam fillings, but not with fish consumption (Table 4).

## Discussion

This study of Hg biomarkers was possible due to the improvements and modifications of the CVAFS method used for determination of total and inorganic Hg in blood, hair and urine. The method provides high sensitivity, low analytical variability, and high accuracy also in the low concentration range. The limits of detection (between 0.01 and 0.09) were about 2–10 times lower than those previously reported [18,22,23,30,34-37]. It can be concluded that our modified analytical method is suitable for the purposes of speciation of Hg in human biological media and for evaluation of the main exposure sources.

The total variability in the different biomarkers measured includes inter-individual differences in the Hg kinetics as well as demethylation of MeHg to IHg during sample treatment and analysis. We have determined the degree of demethylation in our analytical procedure and we conclude that the method results in about 5% demethylation of MeHg, half of it in the solubilisation step if samples are heated during solubilisation (as for hair), and the other half in the analytical step. If solubilisation takes place at room temperature, as for blood, RBC, plasma and urine, the overall demethylation is further reduced, to less than 4%. The acidification of the samples, which was a prerequisite for the extraction procedure and the separation of IHg from MeHg, may be responsible for some of the demethylation during the tests. The demethylation of MeHg in blood during sample preparation and analysis has previously been reported to be 2–3% using acidic digestion [38].

The distribution of OHg between RBC (87%) and plasma (9.6%) was in good agreement with the earlier observations that the major part of MeHg in blood is found in the RBC, bound to haemoglobin [6,7,19]. The inter-individual variation was relatively low (total range about 15%). The average RBC to plasma OHg ratio of 14 found in the present study was between the ratios of 10 [6,7] and 20 [19], previously reported. In those studies, a limited number of volunteers (3 to 15 male and female volunteers) were given oral doses of either a radioactive MeHg salt in solution (about 10 μg Hg; [7]), radioactive MeHg bound to fish muscle protein (about 10 μg Hg; [6]) or a meal of fish containing 18–22 μg Hg/kg b.w. (providing 1400 μg Hg/70 kg man; [19]). The RBC to plasma OHg ratio in the present study was not influenced by the mercury concentrations in blood. Thus, it seems as the distribution of MeHg between RBC and plasma is rather constant over a large range of exposures.

The distribution of IHg between RBC (34%) and plasma (64%) displayed a much larger inter-individual variation (total range about 40–50%) than that of OHg. The RBC-IHg was positively correlated with both P-IHg and RBC-OHg, but P-IHg was not correlated with RBC-OHg. It can be concluded that IHg in RBC is partly emanating from inorganic Hg exposure, mainly Hg^0 ^via amalgam, and partly from MeHg exposure via fish, which has demethylated to IHg in the body and, to some extent, in the analysis (less than 4%). Thus, the variation in MeHg exposure from fish adds to the variation in RBC-IHg, which can partly explain the larger inter-individual variation measured in the distribution of IHg between RBC and plasma. Little is known about the mechanisms involved in the conversion of MeHg to IHg in the human body, and the inter-individual variability. Based on our data, there seems to be little demethylation taking place in the blood (a few percent).

Our data strongly indicates that the small fraction of IHg in hair (about 9%), with relatively small inter-individual variations (CV about 15%) is a result of MeHg exposure and demethylation of MeHg in blood or hair follicles (and in the analysis), rather than a result of IHg exposure. IHg in hair was positively correlated with fish intake, but not with dental amalgam fillings. It was also highly correlated with OHg in blood, RBC and plasma. The hypothesis is further supported by our results in non-fish eating individuals, which showed a positive correlation of MeHg in blood and hair, but no correlation of IHg in blood and hair, despite a very low MeHg exposure (B-MeHg below 1.0 μg/L; [4]). MeHg in hair has been shown to be stable over time [39,40], indicating that demethylation within the hair strand is very limited. However, it should be borne in mind that artificial waving and other hair treatments may reduce Hg concentrations within the hair strand [41]. It has previously been suggested that MeHg is demethylated to inorganic Hg in the cells of the hair follicle [27]. As the IHg fraction in hair was about 9% and since the demethylation of MeHg in the hair analysis is about 5%, the average degree of demethylation in the hair follicles would be on average 4%. Because of the demethylation, THg in hair is a better measure of MeHg exposure than MeHg in hair.

In humans, a frequently cited blood to hair ratio (B-THg:H-THg) evaluated by linear regression is 1:250, however with large inter-study variations (range 140–370; [2,28]). In the present study, the ratio as evaluated by linear regression was 1:254. When evaluating mercury blood to hair ratios by linear regression there is always a positive intercept. The intercept may reflect the different time frames of the integrated exposure as measured in hair and blood, and occasional high MeHg exposure. If the blood to hair ratio of 1:250 is used to calculate B-MeHg from H-THg, B-MeHg will always be underestimated due to the positive intercept. The inter-individual variation in the blood to hair ratio as determined by division was very large. The variability seemed to decrease with increasing B-OHg concentrations (Figure 3), most probably due to more frequent fish consumption and thereby blood concentrations approaching steady state.

Our data shows that IHg in urine reflects the IHg exposure as nearly all Hg in urine (> 98%) was IHg and as IHg in urine did not reflect fish consumption or the OHg concentration in various media. Experimental data report greater concentrations of Hg in kidneys in males than in females exposed similarly [42]. A higher excretion of IHg in urine in women (1.5 μg/L adjusted to specific gravity 1.019 g/mL and 2.1 μg/g creatinine) than in men (0.80 μg/L adjusted to specific gravity 1.019 g/mL and 0.75 μg/g creatinine; p = 0.03) was noted in the present study, despite a similar exposure to IHg as measured by IHg in plasma (0.4 μg/L). However, the sample size was too small to draw any conclusions from those data. Further studies are warranted on gender differences in Hg metabolism and toxicity.

## Conclusion

As expected, fish consumption was positively correlated with THg in blood, RBC, and hair. The use of THg concentration in blood as a proxy for MeHg exposure will give rise to an overestimation of the MeHg exposure, small or large, depending on the exposure to IHg (Hg^2+ ^and Hg^0^). In order to reduce the inter-individual variability it can be recommended to speciate the various forms of Hg in blood when evaluating exposure, dose and risk for health effects. The demethylation taking place during sample preparation and analysis with this method will lead to a small underestimation of the MeHg concentration and an overestimation of the IHg concentration in the sample.

The total Hg concentration in the RBC gives a good measure of the MeHg exposure at low IHg exposure levels. Most of the Hg found in the RBC is in the form of MeHg with small inter-individual variations. Part of the IHg in RBC is emanating from demethylated MeHg, leaving a small fraction of IHg that is the result of IHg exposure.

Using THg concentrations in plasma as a measure of IHg exposure can lead to significant exposure misclassifications. The total concentration of Hg in plasma is associated with both IHg and OHg, with large inter-individual variations in the distribution between RBC and plasma, depending on both the MeHg and IHg exposure.

The THg concentration in hair reflects MeHg exposure at all exposure levels. The small fraction of IHg in hair is most probably emanating from MeHg that was demethylated in the body and during the sample preparation and analysis. IHg in hair was also correlated with fish consumption. THg in hair seems to provide the best measure of long-term average MeHg exposure. THg in urine reflects IHg exposure, also at very low exposure levels. Number of dental amalgam fillings was highly positively correlated with THg in plasma and urine.

## List of abbreviations

Ar Argon

B Blood

B-EVF Blood-Erythrocyte Volume Fraction (haematocrit)

b.w. Body weight

CI Confidence interval

CV Coefficient of variation

CVAFS Cold vapour atomic fluorescence spectrophotometry

H Hair

Hg^0 ^Elemental mercury vapour

Hg^2+ ^Inorganic mercury, ionic form

IHg Inorganic mercury

LOD Limit of detection

MeHg Methylmercury

MIA Multiple-injection analysis

n Number

OHg Organic mercury

P Plasma

RBC Red blood cell

Rec. value Recommended value

THg Total mercury

U Urine

## Competing interests

The author(s) declare that they have no competing interests.

## Authors' contributions

MB participated in the design of the study, performed the data analyses and drafted the manuscript, BL participated in the development of the analytical method and revision of the manuscript, KAB participated in the design of the study and helped to draft the manuscript, BP carried out the mercury analyses, ÖE participated in the development of the analytical method, and MV participated in the design of the study and the critical revision of the manuscript. All authors read and approved the final manuscript.
